# G_673_ could be a novel mutational hot spot for intragenic suppressors of *pheS5* lesion in *Escherichia coli*

**DOI:** 10.1002/mbo3.161

**Published:** 2014-05-08

**Authors:** Thangaraj Ponmani, M Hussain Munavar

**Affiliations:** Department of Molecular Biology, School of Biological Sciences, Centre for Excellence in Genomic Sciences, Centre for Advanced Studies in functional and organismal Genomics, Madurai Kamaraj University [University with Potential for Excellence]Madurai, 625 021, India

**Keywords:** *Escherichia coli*, hot spot, intragenic suppressor, PheRS enzyme, *pheS5*.

## Abstract

The *pheS5* Ts mutant of *Escherichia coli* defined by a G_293_ → A_293_ transition, which is responsible for thermosensitive Phenylalanyl-tRNA synthetase has been well studied at both biochemical and molecular level but genetic analyses pertaining to suppressors of *pheS5* were hard to come by. Here we have systematically analyzed a spectrum of Temperature-insensitive derivatives isolated from *pheS5* Ts mutant and identified two intragenic suppressors affecting the same base pair coordinate G_673_ (*pheS19* defines G_673_ → T_673_; Gly_225_ → Cys_225_ and *pheS28* defines G_673_ → C_673_; Gly_225_ → Arg_225_). In fact in the third derivative, the intragenic suppressor originally named *pheS43* (G_673_ → C_673_transversion) is virtually same as *pheS28*. In the fourth case, the very *pheS5* lesion itself has got changed from A_293_ → T_293_ (named *pheS40*). Cloning of *pheS*^*+*^, *pheS5, pheS5-pheS19, pheS5-pheS28* alleles into pBR322 and introduction of these clones into *pheS5* mutant revealed that excess of double mutant protein is not at all good for the survival of cells at 42°C. These results clearly indicate a pivotal role for Gly_225_ in the structural/functional integrity of alpha subunit of *E. coli* PheRS enzyme and it is proposed that G_673_ might define a hot spot for intragenic suppressors of *pheS5*.

## Introduction

Aminoacyl-tRNA synthetases (aaRS) play a pivotal role in translation process in all living systems. They are mainly implicated in fidelity during translation by way of attaching correct amino acid to the cognate t-RNAs (Ling et al. 2007). Although it was originally conceived that aaRS might have restricted function in aminoacylation during translation, now it is becoming increasingly evident that many synthetases possess more than one function; they are involved in cellular fidelity, t-RNA processing, RNA splicing, RNA trafficking, apoptosis, and transcription control as well as translation control (Martinis et al. 1999a,b). Multifaceted nature of aaRS has been well established in both prokaryotic and eukaryotic systems (Romby and Springer 2003; Park et al. 2005; Bori-Sanz et al. 2006; Guo et al. 2010; Smirnova et al. 2012; Guo and Schimmel 2013). Although *Escherichia coli* phenylalanyl-tRNA Synthetase comes under class II based on structural considerations, it charges the amino acid at the 2′ hydroxyl group of the adenosine of tRNA like a class I enzyme (Schimmel 1987). The *pheS* and *pheT* genes that code for *α* and *β* subunits of phenylalanyl-tRNA synthetase define an operon with the direction of transcription being *pheS* to *pheT* (Springer et al. 1982; Berlyn 1998). It is well known that this synthetase functions as a hetero tetramer and it is unique among the tRNA synthetases perhaps other example being glycyl-tRNA synthetase (Martinis and Schimmel 1996). Of more particular relevance to this article is the work done in our laboratory on genetics of *fit* loci, genes implicated in transcription control in *E. coli*. It has been reported that a temperature-sensitive but primarily transcription-defective mutant (*fitA76*) bears two lesions, one located in *pheS* gene and the other, named *fit95*, located in *pheT*. The *pheS* mutation is virtually same as that present in temperature-sensitive but translation-defective mutant *pheS5* and bears a G_293_ → A_293_ transition. The other mutation *fit95*, by itself exhibits interesting phenotypes, but has to be present along with *pheS5* in order to elicit the phenotype characteristic of *fitA76* mutant. Moreover, two suppressors of *fitA76* namely *fitA24* and *fitB* have also been isolated and characterized. Even more interestingly, four rifampicin-resistant *rpoB* mutations have been identified which were capable of modulating phenotype of *fit* mutant in an allele-specific manner. All these results culminated in the notion that both *α* and *β* subunits of phenylalanyl-tRNA synthetase also function as Accessory Transcription Factors (FitA and FitB) and are involved in selective gene expression in *E. coli* (Jabbar and Jayaraman 1976, 1978; Dass and Jayaraman 1985a,b, 1987; Munavar and Jayaraman 1987, 1993; Munavar et al. 1993; Ramalingam et al. 1999; Vidya et al. 2006; reviewed by Jayaraman 1994). Considering the emerging trends about the expanding functions of synthetases as cited above, our view that PheRS enzyme of *E. coli* can also function as Accessory Transcription Factor (FIT) does not look like a far-fetched view. It is this work of ours that prompted us to independently investigate the potential suppressors of temperature-sensitive primarily translation-defective *pheS5* mutant (Eidlic and Neidhardt 1965; Kast et al. 1992). We hereby report the characterization of a collection of Temperature-insensitive derivatives isolated from *pheS5* mutant and the analyses reveal that base pair coordinate G673 of *pheS* gene might define a potential hot spot for intragenic suppressors for *pheS5*. We propose that relevant amino acid coded by the codon affected by these mutations could be of immense importance in structural and functional integrity of *α* subunit of PheRS enzyme.

## Experimental Procedures

### Bacterial strains, bacteriophage, and plasmids

All *E. coli* strains used are derivatives of *E. coli* K12. P1 *vir*, originally from Dr. N Willetts, U.K. and maintained locally. Since most of the strains are Ts and their derivatives, depending on the nature of the experiment, the strains were grown at either 30°C or 42°C. Given in Tables S1 and S2 are the strains, plasmids used, and clones constructed for this study. Genetic Nomenclature is given according to Demerec et al. (1966).

### Media, enzymes and biochemicals

Conventional LB medium and M9 minimal medium were used according to Miller (1972, 1992). Reagent grade materials used for preparation of various media, solutions, and buffers were purchased mainly from Hi–Media-India. Antibiotics and other fine chemicals were purchased from Sigma Company (Sigma Chemicals, St. Louis, MO). Restriction Enzymes, T4 DNA Ligase, dNTPs, and *pfu* polymerase were purchased from MBI-Fermentas, *Genetix*, Germany.

### Methods

The molecular genetic techniques used in this study were mostly as described in Miller (1972, 1992) or with minor modifications. Most of the recombinant DNA techniques employed were according to Sambrook et al. (1989); Sambrook and Russel (2001). P1-mediated mobilization of *pheS* allele(s) was carried out essentially by taking the advantage of *pps*::Tn*10* marker which is linked with *pheS* (60% cotransduction).

### DNA isolation, polymerase chain reaction amplification, and sequencing

A simple and rapid method described by Chen and Kuo (1993) was followed to isolate genomic DNA from relevant *E. coli* strains. The following primers were used to amplify the *pheS* fragment *pheS* Forward primer 5′ATTGACTTTTATCGCCGTAGC3′ and *pheS* Reverse primer 5′TTTGAGGAAACGCAGATCG 3′, with the cyclic condition: Initial Denaturation 94°C for 5 min; Denaturation 95°C for 45 sec; Primer annealing 61°C for 45 sec; Extension 72°C for 2 min; Final extension 72°C for 5 min with *pfu* polymerase. The Sequencing of amplified *pheS* (∼1.5 kb fragment) was done by Chromous Biotech, Pvt. Ltd. Bangalore, India.

### Molecular cloning of relevant *pheS* alleles

Polymerase chain reaction (PCR)-based cloning strategy was followed to clone the relevant *pheS* alleles (Sambrook et al. 1989; Sambrook and Russel 2001). The following primers F1 and R were used to amplify and then clone the *pheS* region with Native and Alternate promoters; Forward primer F1 5′CGC**AAGCTT**TCGTTTCAACGCC3′ and Reverse primer R **5**′C**GGATCC**TTATTTA AACTGTTTGAG3′. The following primers F2 and R were used to amplify the *pheS* region only with Alternate promoter of *pheST* operon Forward primer F2 5′CGC**AAGCTT**TTTTGAAGAG TACCAA 3′and Reverse primer R 5′C**GGATCC**TTATTAAACTGTTTGAG3′. Forward primers F1 and F2 were designed with *Hind*III site and the Reverse primer R was designed with *BamH*I restriction site in order to enable directional cloning of each of the above amplicon to generate the relevant clone.

### Estimation of relative viability

Necessary dilutions of the cultures of the relevant strains were plated on LB agar plates (drugs were included if necessary) and one set was incubated at 30°C and the other at 42°C as the case may be. Colonies were counted after 36–48 h of incubation. Relative viability (RV) refers to CFU mL at 42°C/CFU mL at 30°C.

## Results

### Isolation of temperature-insensitive derivatives of *pheS5* Ts mutant

NP37 is the Classical temperature-sensitive translation-defective mutant which harbors *pheS5* mutation defined by a G_293_ → A_293_ transition in *pheS* gene (Kast et al. 1992). This strain grows normally at 30°C and does not grow at 42°C and the RV of NP37 at 42°C is in the order of 10^−8^ (see Table 1). In order to isolate potential stable Temperature-insensitive derivatives of *pheS5* mutant, 1ml of overnight grown saturated culture of NP37 *pps::Tn10* strain was taken, spun down, and the pellet was suspended in saline and spread on LB plate containing tetracycline (10 *μ*g mL^−1^) and incubated at 42°C. Six independent experiments were done to avoid getting siblings and a total of 58 Temperature-insensitive (Ts^+^) derivatives were isolated. The stability of all the 58 Ts^+^ derivatives were checked by repeatedly growing them for several generations at 30°C and replica patching them at 30°C and 42°C. During our analyses, all the 58 Ts^+^ derivatives were found to be very stable.

**Table 1:** Relative viability of relevant strains

	Promoter(s) and *pheS* alleles present in relevant plasmid clones	*Relative viability 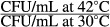
Strain/Relevant Genotype/Phenotype		
NP37 *pheS5* Ts	NA	0.91 × 10^−8^
Ts^+^ derivative 1	NA	0.94
Ts^+^ derivative 2	NA	1.00
Ts^+^ derivative 3	NA	1.00
Ts^+^ derivative 19	NA	0.90
Ts^+^ derivative 28	NA	1.04
Ts^+^ derivative 37	NA	0.87
Ts^+^ derivative 40	NA	0.90
Ts^+^ derivative 43	NA	0.94
Ts^+^ derivative 50	NA	1.02
Ts^+^ derivative 55	NA	1.02
Strain, Genotype/Plasmid present		
NP37 *recA::cam*/pTPMS^+^	NP and AP- *pheS*^+^	0.05
NP37 *recA::cam*/pTPMS5	NP and AP-*pheS5*	<0.24 × 10^−8^
NP37 *recA::cam*/pTPMS519	NP and AP- *pheS5*-*pheS19*	2 × 10^−4^
NP37 *recA::cam*/pTPMS528	NP and AP-*pheS5*-*pheS28*	4.7 × 10^−4^
NP37 *recA::cam*/pBR322 vector-control	Nil	<0.17 × 10^−8^
NP37 *recA::cam*/pTPMS^+^A	AP-*pheS*^+^	1.57
NP37 *recA::cam*/pTPMS5A	AP-*pheS5*	<2 × 10^−8^
NP37 *recA::cam*/pTPMS519A	AP- *pheS5*-*pheS19*	5 × 10^−4^
NP37*recA::cam*/pTPMS528A	AP- *pheS5*-*pheS28*	5 × 10^−5^

* Relative viability refers to average values obtained in three independent experiments. NA, not applicable; NP, native promoter (Fayat et al. 1983); AP, alternate promoter (Kamalakar 2006). For further details see text and methods.

### The suppressors of *pheS5* may be tightly linked to *pheS5*

Since the 58 Ts^+^ derivatives were isolated from six independent experiments, as stated above we would have avoided siblings. It is also possible that some of the temperature-insensitive derivatives could be True revertants in which the *pheS5* (G_293_ → A_293_) lesion could have reverted back to G_293_ itself and thus giving rise to Ts^+^ phenotype. Some others could possibly be pseudorevertants and such pseudorevertants are expected to retain *pheS5* lesion (G_293_ → A_293_ in *pheS*) and would have acquired another mutation as a suppressor. We are compelled to make a model that more than one mutation (as a suppressor) might not have emerged from our selection as they were isolated as spontaneous revertants and not by using a mutagen. Owing to the uncertainties pertaining to map position of the suppressor allele(s) we initially did Genetic analysis. For this purpose, P1 lysates were made on all the 58 Ts^+^ derivatives and the lysates were independently used to transduce the linked *pps::Tn10* marker into a *pps*^*+*^ *pheS*^*+*^ strain MG1655. From all the 58 different transductional crosses, the Tet^R^ (*pps::Tn10*) transductants were obtained on selective plates containing LB+Tet+citrate (see Methods). From each cross, 100 Tet^R^ transductants were taken and replica patched at 30°C and 42°C. Among the 58 transductional crosses not even from a single cross we could get a single transductant exhibiting a Ts phenotype. Evidently, in all crosses, all the checked transductants were temperature insensitive. These results strongly support the notion that all the Ts^+^ derivatives could be true revertants. If some were to be pseudorevertants then, the relevant suppressor in such pseudorevertant alone might not confer a Ts phenotype (See below) and it should be very tightly linked to *pheS5* itself.

### Quantification of suppression of *pheS5* Ts phenotype in selected 10 derivatives

Among the 58 revertants, 10 were characterized in detail. First, in order to have a clue about the nature of suppressor (True, Pseudo) in these selected 10 derivatives, we studied the Relative level of suppression of Ts phenotype (RV). In these 10 derivatives some times, some pseudorevertants might exhibit a stable phenotype in qualitative analysis but RV values may not be 1. Therefore, all the 10 Ts^+^ (chosen) isolates were grown overnight in LB+Tet and relevant dilutions were plated at 30°C and 42°C. The RV values were in the order of ∼1 in all the revertants (Table 1) and this suggests that among the 10, if any or some were to be pseudorevertants, then the level of suppression of *pheS5*Ts mutant phenotype due to the corresponding suppressor allele should be of very good degree.

### Genetic mapping of suppressor(s) of *pheS5*

In any genetic screen pertaining to isolation of revertants for a mutant, one can always expect intragenic and extragenic suppressors and also true revertants. While extragenic suppressors are predominantly associated with components of interacting system involved in a particular function/pathway (Garza et al. 1996; Rokop and Grossman 2009) intragenic suppressors are the ones which help us to know the functional domain(s)/critical amino acids in the relevant protein (Helinski and Yanofsky 1963; Davis et al. 1999; Shiomi et al. 2002). During the isolation of Ts^+^ derivatives of *pheS5*, we can expect the suppressors from *pheS* gene itself (intragenic) to restore the activity of PheS5 mutant protein at 42°C or the suppressors could be extragenic that is from its functional partner *pheT* (enabling the *pheT* mutation to form active PheRS tetrameric complex with mutated PheS5 protein) or could be in some other gene(s), the product(s) of which might function in a hitherto unreported manner so as to restore the normal function of the mutant PheS5 product. In all the above-mentioned cases, by introducing the relevant suppressor allele together with *pheS5* (since it is linked with *pheS5* and difficult to separate; see above) into an authentic *pheS5* mutant with linked marker, one can ascertain the position of the suppressor. With this in mind, P1 lysates made on these 10 Ts^+^ derivatives were used to transduce the linked *pps::Tn10* into a *pps*^+^ *pheS5* strain and Tet^R^ transductants were obtained in LB+Tet+citrate plates (See Methods). In each cross ∼100 transductants were checked for the Temperature-insensitive (Ts^+^) phenotype. In almost all cases more than 70% transductants became Ts^+^ and grew very well at 42°C (Table 2). These results clearly indicate that the lesion responsible for the Ts^+^ nature of the revertants very well cotransduce with *pps::Tn10* and in all likelihood the suppressor in pseudorevertants, if any, should lie between *pheS5* and *pps* and must be tightly linked to *pheS5* itself and sequence analyses reported below confirm this view.

**Table 2:** The % Cotransduction of Ts^+^ Phenotype among Tet^R^ (*pps::Tn10)* transductants obtained in the 10 different transductional crosses in relevant strains

Donor	Recipient	Selected marker/Character	Unselected phenotype	% Cotransduction
P1/Ts^+^ derivative 1	NP37 Ts *pheS5 pps*^*+*^	*pps::Tn10* (Tet^R^)	Ts^+^	89.1 (114/128)
P1/Ts^+^ derivative 2	NP37 Ts *pheS5 pps*^*+*^	*pps::Tn10* (Tet^R^)	Ts^+^	85.6 (154/180)
P1/Ts^+^ derivative 3	NP37 Ts *pheS5 pps*^*+*^	*pps::Tn10* (Tet^R^)	Ts^+^	80.5 (89/112)
P1/Ts^+^ derivative 19	NP37 Ts *pheS5 pps*^*+*^	*pps::Tn10* (Tet^R^)	Ts^+^	86.5 (96/111)
P1/Ts^+^ derivative 28	NP37 Ts *pheS5 pps*^*+*^	*pps::Tn10* (Tet^R^)	Ts^+^	80.95 (51/63)
P1/Ts^+^ derivative 37	NP37 Ts *pheS5 pps*^*+*^	*pps::Tn10* (Tet^R^)	Ts^+^	87.7 (164/187)
P1/Ts^+^ derivative 40	NP37 Ts *pheS5 pps*^*+*^	*pps::Tn10* (Tet^R^)	Ts^+^	82.9 (145/175)
P1/Ts^+^ derivative 43	NP37 Ts *pheS5 pps*^*+*^	*pps::Tn10* (Tet^R^)	Ts^+^	84.5 (163/193)
P1/Ts^+^ derivative 50	NP37 Ts *pheS5 pps*^*+*^	*pps::Tn10* (Tet^R^)	Ts^+^	89.3 (108/121)
P1/Ts^+^ derivative 55	NP37 Ts *pheS5 pps*^*+*^	*pps::Tn10* (Tet^R^)	Ts^+^	87 (147/169)
P1/*pps::Tn10 pheS*^*+*^	NP37 Ts *pheS5 pps*^*+*^	*pps::Tn10* (Tet^R^)	Ts^+^	80.87 (93/115)

WT *pheS*^*+*^ linked *pps::Tn10* was also used for transductional cross as control.

### Sequence analyses of the *pheS* region from all the selected Ts^+^ derivatives: identification of a potential hot spot for intragenic suppressors of *pheS5*

To prove the veracity of the view derived from genetic analyses, we sequenced the relevant *pheS* region of all the 10 Ts^+^ derivatives by PCR-based cyclic sequencing (See Methods). Sequence analyses were done with NCBI Blast to know the base change(s) in *pheS* in all the 10 Ts^+^ derivatives (Table 3). The sequence analyses of the 10 Ts^+^ derivatives and comparison of the same with *pheS5/pheS*^+^ sequences clearly revealed that in three Ts^+^ derivatives (19, 28, and 43) *pheS5* lesion is intact and they have also acquired a suppressor mutation. It is very interesting to realize the fact that all the three Ts^+^ derivatives bear the suppressor mutation at the same base pair coordinate 673 of *pheS*. In fact, in two of the three cases (Ts^+^ derivatives 28 and 43*)* the base change at the position 673 is virtually same (G_673_ → C_673_; Gly_225_ → Arg_225_). In the third case, the base change is G_673_ → T_673_ (Ts^+^ derivative 19), which changed the amino acid from Gly_225_ to Cys_225_. On the basis of these results we are compelled to make a proposal that G_673_ might define a potential hot spot for intragenic suppressor of *pheS5*. In one other revertant, the base position 293 affected in *pheS5* namely G_293_ → A_293_ got changed to T_293_ (Ts^+^ derivative 40) leading to Asp_98_ to Val_98_ amino acid change. We found that the rest of the other Ts^+^ derivatives were true revertants, in which G_293_ → A_293_ has got reverted back to G_293_ itself (Ts^+^ derivatives 1, 2, 3, 37, 50, and 55) The suppressor mutation in Ts^+^ derivatives 19, 28, and 43 were named as *pheS19, pheS28*, and *pheS43,* respectively. In the revertant 40, we have named the lesion as *pheS40*. It should be noted that *pheS28* and *pheS43* define the same allele.

**Table 3:** Summary of sequence changes and corresponding amino acid changes in relevant Ts^+^derivatives

	Lesion present at *pheS5* position		Lesions present at the position of the suppressor mutation		
			
Strain	Base change	Aminoacid change	Base change	Aminoacid change	Inference/Source
*pheS*^*+*^ (WT)	G_293_ (WT)/NR	Gly (WT)/NR	NR	NR	Ramalingam et al. (1999) and also this work
NP37 *pheS5*	G_293_ → A_293_	Gly_98_ → Asp_98_	NR	NR	Kast et al. (1992); Ramalingam et al. (1999)
Ts^+^ derivative 1	A_293_ → G_293_	Asp_98_ → Gly_98_	NF	NF	True revertant; this work
Ts^+^ derivative 2	A_293_ → G_293_	Asp_98_ → Gly_98_	NF	NF	True revertant; this work
Ts^+^ derivative 3	A_293_ → G_293_	Asp_98_ → Gly_98_	NF	NF	True revertant; this work
Ts^+^ derivative 19	G_293_ → A_293_	Gly_98_ → Asp_98_	G_673_ **→** T_673_	Gly_225_ **→** Cys_225_	Pseudo-revertant; this work
Ts^+^ derivative 28	G_293_ **→** A_293_	Gly_98_ **→** Asp_98_	G_673_ **→** C_673_	Gly_225_ **→** Arg_225_	Pseudo-revertant; this work
Ts^+^ derivative 37	A_293_ **→** G_293_	Asp_98_ **→** Gly_98_	NF	NF	True revertant; this work
Ts^+^ derivative 40	A_293_ **→** T_293_*	Asp_98_ **→** Val_98_	NF	NF	Pseudo-revertant; this work
Ts^+^ derivative 43	G_293_ **→** A_293_	Gly_98_ **→** Asp_98_	G_673_ **→** C_673_	Gly_225_ **→** Arg_225_	Pseudo-revertant; this work
Ts^+^ derivative 50	A_293_ **→** G_293_	Asp_98_ **→** Gly_98_	NF	NF	True revertant; this work
Ts^+^ derivative 55	A_293_ **→** G_293_	Asp_98_ **→** Gly_98_	NF	NF	True revertant; this work

NR, not relevant; NF, not found.

* The base change is A **→** T but at the same position 293.

### Isopropyl *β*-D-1-thiogalactopyranoside-induced expression of *β*-galactosidase in *pheS5* mutant and in the suppressed derivatives

**Figure 1:**
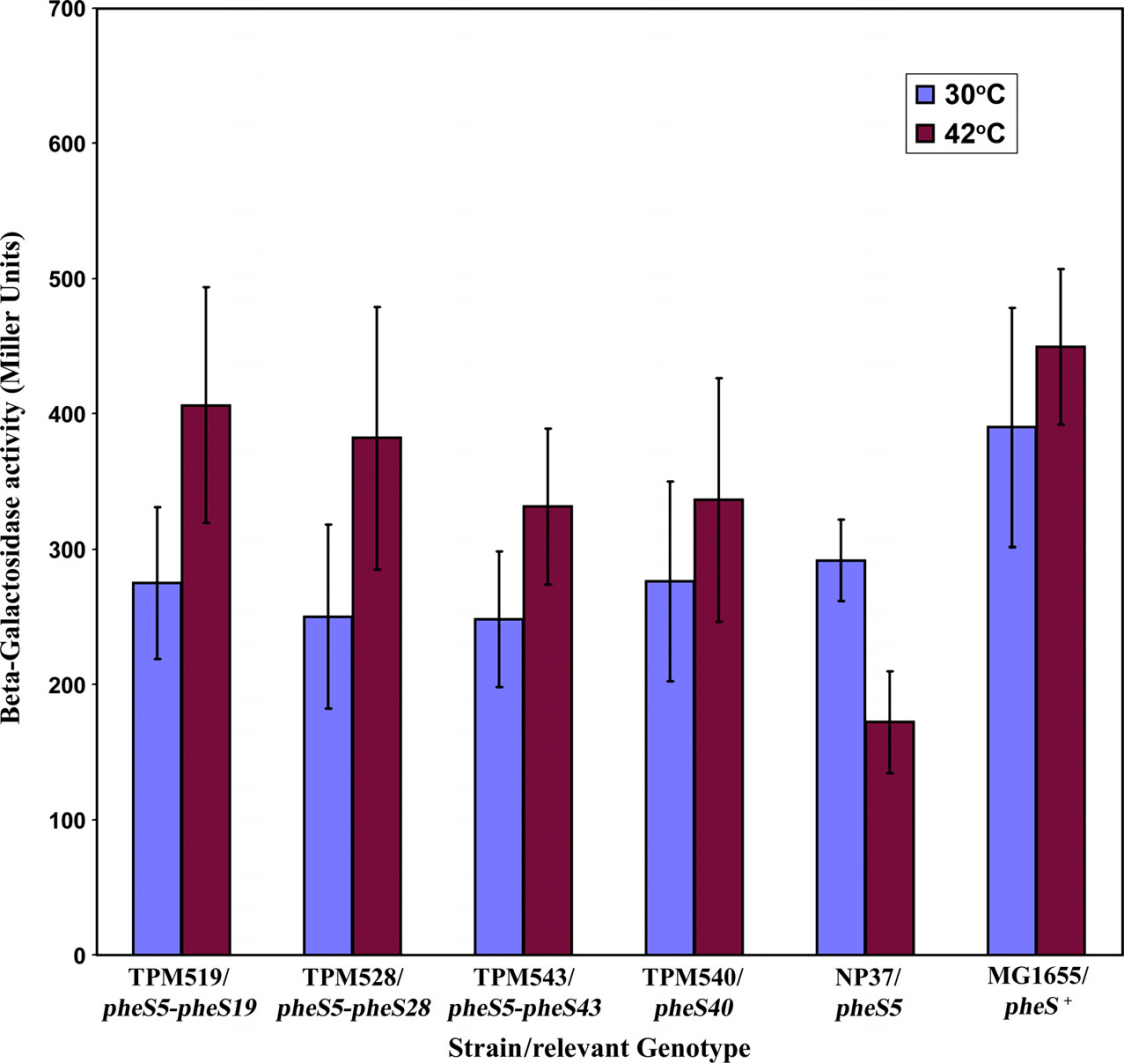
Induced expression of *β*-galactosidase in MG1655 *pheS*^+^, NP37 *pheS5* (Ts) and its suppressed Ts^+^ derivatives. SEM-standard error mean bar (calculated from three independent experiments) was also indicated.

It is well known that *pheS5* mutation leads to primary defect in translation at 42°C (Kast and Henneck 1991; Ramalingam et al. 1999; Sudha et al. 2001). In the pseudorevertants reported herein, one would logically expect restoration of translational defect to a considerable extent at 42°C because their RV values were in the order of ∼1. One can indirectly study the same by way of measuring the expression of the candidate gene *lacZ* that is *β*-Galactosidase levels at 30°C and 42°C in *pheS5* as well as in the corresponding Ts^+^ derivatives. Figure 1 clearly indicates that in *pheS5* mutant, after shifting the culture from 30°C to 42°C within 15 min. there is about 50% reduction in the expression of *β*-Galatosidase at 42°C as compared to that at 30°C and this is in accordance with the expectation. In Ts^+^ derivatives with suppressor of *pheS5*, the *β*-Galatosidase activity at 42°C was not affected at all upon shift to 42°C as in the WT strain. These results reinforce the earlier conclusion (see above) that the suppression due to intragenic suppressor is strong in all the four cases. The Ts^+^ derivatives 19, 28, 43, and 40 bearing *pheS5*-*pheS19, pheS5*-*pheS28, pheS5*-*pheS43*, and *pheS40* mutations were named as TPM519, TPM528, TPM543, and TPM540, respectively.

### Molecular cloning of relevant *pheS* allele(s) with native and alternate promoter alone

Next we wanted to know the effect of *pheS5*-*pheS19* and *pheS5*-*pheS28* alleles in *trans* (when cloned in a plasmid) in a *pheS5* Ts mutant. It is needless to clone the corresponding intragenic suppressor alleles alone (*pheS19* and *pheS28*) because intragenic suppressor alleles alone usually do not complement the mutant phenotype for which they have been isolated as suppressors, in this case *pheS5*. In two different mutations, if one is an intragenic suppressor for the other they can suppress only in *cis* and will fail to complement in *trans*. By and large this is expected because both mutations will independently code for a nonfunctional protein. We believe this statement will hold water in our case also. However, if the *pheS5* allele is cloned with the relevant suppressor alleles in a plasmid (*pheS5-pheS19* and *pheS5-pheS28*) then seeing their effect in a *pheS5* mutant will be really interesting because it would give a clue about whether the double mutant PheS protein (PheS5-PheS19 or PheS5-PheS28) will enable the formation of active PheRS complex despite the presence of single PheS5 mutant protein encoded by the *pheS5* allele of the chromosome. The original promoter of *pheST* operon was initially identified by Fayat et al. (1983), namely Native promoter (hereinafter referred as NP) located at 368 nucleotides upstream of ATG of *pheS* (TTCAATA). The transcription originating from this promoter is subjected to an attenuation control and several reports are available on this aspect (Trudel et al. 1984; Springer et al.^1985^; Mechulam et al. 1987; Gollnick and Babitzke 2002). It is Kamalakar during his Ph.D., thesis work from this laboratory (Kamalakar 2006; B. P. Kamalakar, M. H. Munavar, and R. Jayaraman, unpubl. data), who constructed several deletions in region of *pheST* operon, particularly just upstream to ATG of *pheS*. During his analyses, he identified that the sequence “TCTAAGT” located about 45bp upstream of ATG of *pheS* can indeed function as a promoter and named the same as “Alternate promoter” (hereinafter referred as AP). Several lines of evidences do support the notion that the sequence proposed to function as AP might be of importance in the regulation of expression of *pheST* operon (Kamalakar 2006; B. P. Kamalakar, M. H. Munavar, and R. Jayaraman, unpubl. data).

Cloning of all the relevant alleles was done based on PCR cloning strategy with appropriate forward and reverse primers with *Hind*III and *BamH*I flanking restriction sites. In order to enable the cloning of *pheS5*-*pheS19, pheS5*-*pheS28, pheS5*, and *pheS*^+^ alleles, all the above-mentioned relevant *pheS* regions with indicated *pheS* alleles (∼1.5 kb) were first amplified using the relevant primers and then cloned in pBR322 with both Native (which includes Alternate promoter as well) and with Alternate promoter alone. The presence of *pheS* insert in each clone was then confirmed by double digestion with *Hind*III and *BamH*I restriction enzymes (data not shown). *pheS* clones bearing relevant *pheS* alleles with both Native and Alternate Promoters were named as follows pTPMS519 (*pheS5*-*pheS19*), pTPMS528 (*pheS5*-*pheS28*), pTPMS5 (*pheS5*), and pTPMS^+^ (Wild Type *pheS*^+^). *pheS* clones bearing Alternate promoter alone were named as pTPMS519A (*pheS5*-*pheS19*), pTPMS528A (*pheS5*-*pheS28*), pTPMS5A (*pheS5*), and pTPMS^+^A (Wild Type *pheS*^+^) respectively.

### Extent of complementation/suppression of Ts phenotype of *pheS5* by relevant *pheS* clones

**Figure 2:**
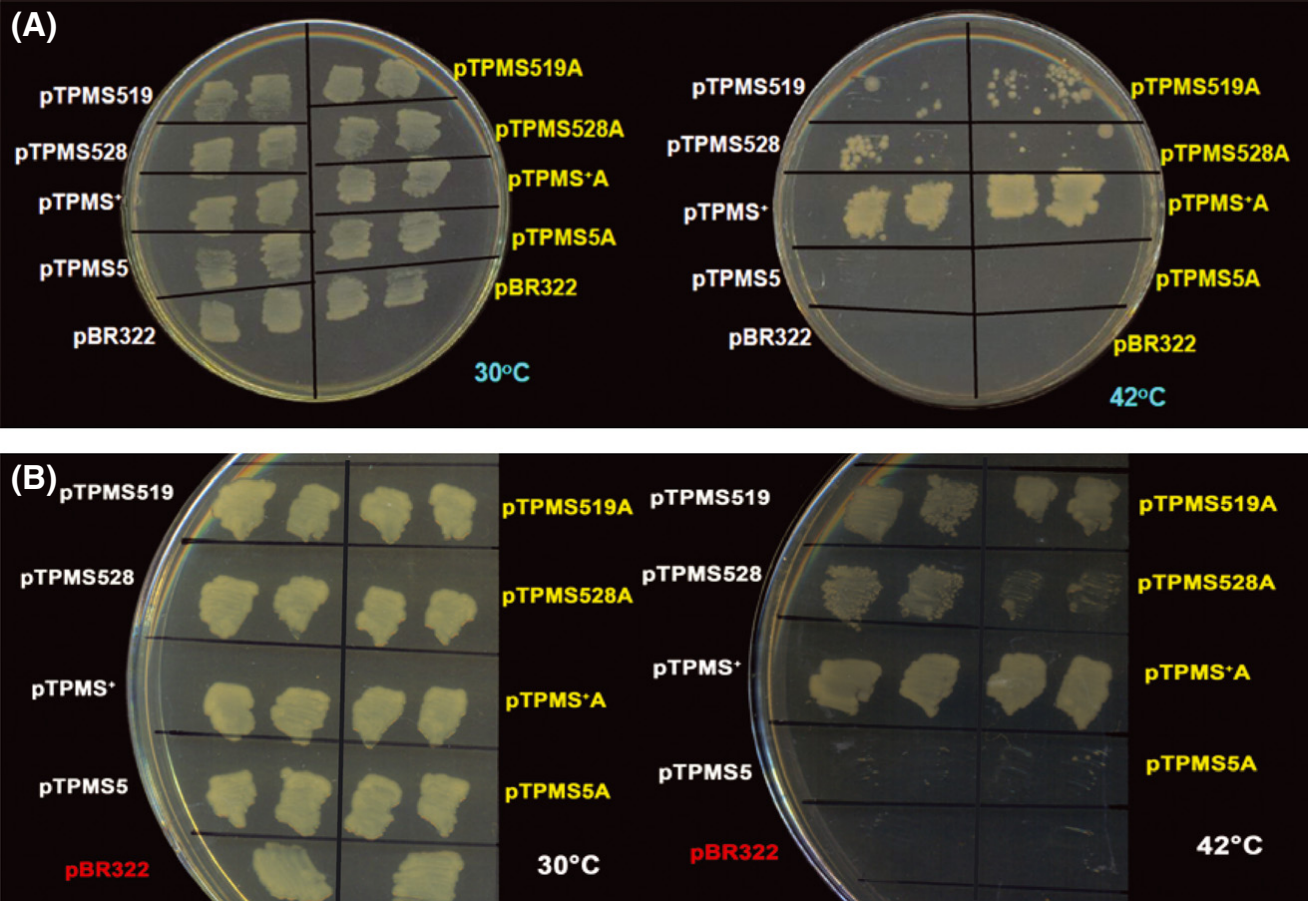
(A) Complementation analyses of the Ts phenotype of *pheS5* by relevant clones: Nature of growth of NP37 (*pheS5*) Ts mutant harboring relevant plasmid clone at 30°C and 42°C when patched with ∼10^5^ cells, from diluted culture (Table 1). (B) Complementation analyses of the Ts phenotype of *pheS5* by relevant clones: Nature of growth of NP37 (*pheS5*) Ts mutant harboring relevant plasmid clone at 30°C and 42°C when we patched directly from well-grown colonies/patches (Table 1).

In order to study the extent of complementation/suppression of *pheS5* Ts phenotype by these plasmids, all the clones bearing relevant *pheS* regions were transformed into NP37 (*pheS5) recA::cam* mutant to know their effect in *trans* and the extent of restoration of the functional PheRS. pBR322 plasmid vector was used as control. All the relevant clone-bearing strains with appropriate controls were grown at 30°C in LB+Amp plates. Appropriate dilutions of the cultures were plated at 30°C and 42°C in LB+Amp plates. After 24 h incubation CFU/mL at 30°C and at 42°C were calculated and then the RV values were calculated (Table 1). Shown in Figure 2A and B is nature of growth of *pheS5* mutant with all these clones.

As could be seen from the results, in NP37 *pheS5* genetic background, the plasmid clone-bearing *pheS*^*+*^ allele with AP (pTPMS^+^A) complemented the Temperature-sensitive phenotype much better when compared with the *pheS*^*+*^ clone (pTPMS^+^). Complementation of Temperature-sensitive phenotype due to *pheS5* by Plasmid clones (either cloned with NP or cloned with AP alone) bearing either *pheS5-pheS19* alleles (pTPMS519 and pTPMS519A) or *pheS5-pheS28* alleles (pTPMS528 and pTPMS528A) in NP37 genetic background is poor.

### Genetic evidence that both *pheS19* and *pheS28* mutations either do not confer Ts phenotype or could be lethal when present alone

**Figure 3:**
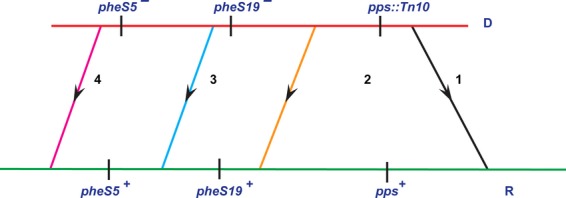
Schematic illustration of the P1 transductional crosses involving TPM519 (D) and MG1655 (R). Note: In this cross TPM519(*pheS5*^*−*^ *pheS19*^*−*^ *pps::Tn10*) was used as donor (D) and *pheS5*^+^*pheS19*^+^*pps*^+^MG1655 served as recipient (R). The first cross-over (1) is shown as a black line and the second cross-overs are shown in coloured lines. For other details, see Tables 4 and 5.

Initial genetic experimentation with 58 Ts^+^ derivatives indicated that the suppressor mutation in relevant pseudorevertants by itself may not confer a Ts phenotype (see above). But this view should be treated conjectural because, in each case/cross in which Ts^+^ derivatives were served as Donor and MG1655 *pps*^*+*^ *pheS*^*+*^ served as Recipient, among Tet^R^ transductants we tested only 100 colonies and did not get even a single Ts transductant (see above). However, 100 colonies might not be a sufficient number if the recombinational separation between *pheS5* and each of the intragenic suppressors (*pheS19* or *pheS28*) is less than 1%. Based on sequence analysis, the distance between *pheS5* and the intragenic suppressors (*pheS19, pheS28*) is exactly 380 base pairs. Owing to such a tight linkage between *pheS5* and the relevant intragenic suppressors *pheS19* or *pheS28*, screening 100 colonies in the above-mentioned crosses might not have given the correct view about the phenotype of suppressor mutations that is whether *pheS19/pheS28* by themselves confer any selectable phenotype or not. Perhaps one has to screen thousands of colonies in such transductional crosses so that the recombinational separation between the two mutations is possible. Such recombinational separation will allow only the coinheritance of relevant intragenic suppressor with selected marker. It is needless to study that the phenotype of *pheS43* since it is same as *pheS28*. In case of *pheS40* it cannot be Ts since the base change is at position 293 itself, and hence, it was not done. Therefore, to know the nature of phenotype due to *pheS19* and *pheS28* mutations, the following was done. P1 lysates were made on the strains TPM519 (*pheS5*-*pheS19*) and TPM528 (*pheS5-pheS28*) and both were independently used to transduce the linked *pps::Tn10* marker into a *pheS*^*+*^ *pps*^*+*^ WT strain and the Tet^R^ transductants were obtained on selective plates containing LB+Tet+citrate. After segregation of transductants, we looked for Ts colonies among Tet^R^ transductants and the results obtained from these crosses clearly indicate that among the 1174 Tet^R^ transductants checked with TPM519 strain as donor not even a single transductant was found to be Ts (Fig. 3, Tables 4 and 5). This is true in the case of *pheS28* mutation also. Among the 1097 Tet^R^ transductants checked not even a single one was found to be Ts when we used TPM528 as Donor (Table 5). Therefore, we are compelled to make a model that either the suppressor mutations (*pheS19* and *pheS28*) by themselves do not confer a Ts phenotype or they will be lethal when present alone.

**Table 4:** Predicted phenotypes and genotypes of Tet^R^ (*pps::Tn10*) transductants expected out of the transductional cross depicted in the Figure 3

Serial No.	Interval of the second crossover	Predicted genotype of the *pps::Tn10* (Tet^R^) transductant	Expected phenotype	Inference/Comments
1	*pheS19*^*−*^–*pps::Tn10*	*pheS5*^*+*^ *pheS19*^*+*^ *pps::Tn10*	Ts^+^	Ts^+^ since *pheS*^*+*^ wild type
2	*pheS5*^*−*^–*pheS19*^*−*^	*pheS5*^*+*^ *pheS19*^*−*^ *pps::Tn10*	?	may or may not confer Ts phenotype and depends purely on the phenotype of *pheS19*^*−*^ allele
3	Beyond *pheS5*^*−*^ to its left	*pheS5*^*−*^ *pheS19*^*−*^ *pps::Tn10*	Ts^+^	Should be Ts^+^ since *pheS19*^*−*^ suppresses *pheS5*^*−*^ Ts phenotype

? indicates that expected phenotype could be Ts or Ts^+^.

**Table 5:** Outcome of the transductional crosses (depicted in the Fig. 3), showing the phenotype of Tet^R^ (*pps::Tn10)* transductants

Ex. No.	Donor/relevant genotype	Recipient/relevant genotype	Selected marker/character	Unselected phenotype	Frequency (Ts/Tet^R^)	Total (Ts/Tet^R^)
1	P1/TPM519 (*pheS5-pheS19 pps::Tn10*)	MG1655 *pheS*^*+*^ *pps*^*+*^	*pps::Tn10* (Tet^R^)	Ts	0/625	0/1174
2	P1/TPM519 (*pheS5-pheS19 pps::Tn10*)	MG1655 *pheS*^*+*^ *pps*^*+*^	*pps::Tn10* (Tet^R^)	Ts	0/549	
1	P1/TPM528 (*pheS5-pheS28 pps::Tn10*)	MG1655 *pheS*^*+*^ *pps*^*+*^	*pps::Tn10* (Tet^R^)	Ts	0/636	0/1097
2	P1/TPM528 (*pheS5-pheS28 pps::Tn10*)	MG1655 *pheS*^*+*^ *pps*^*+*^	*pps::Tn10* (Tet^R^)	Ts	0/461	

NP, native promoter; AP, alternate promoter (Kamalakar 2006).

### Mapping of mutated amino acid residue on a three-dimensional structural model of *E. coli* PheRS (EcPheRS)

**Figure 4:**
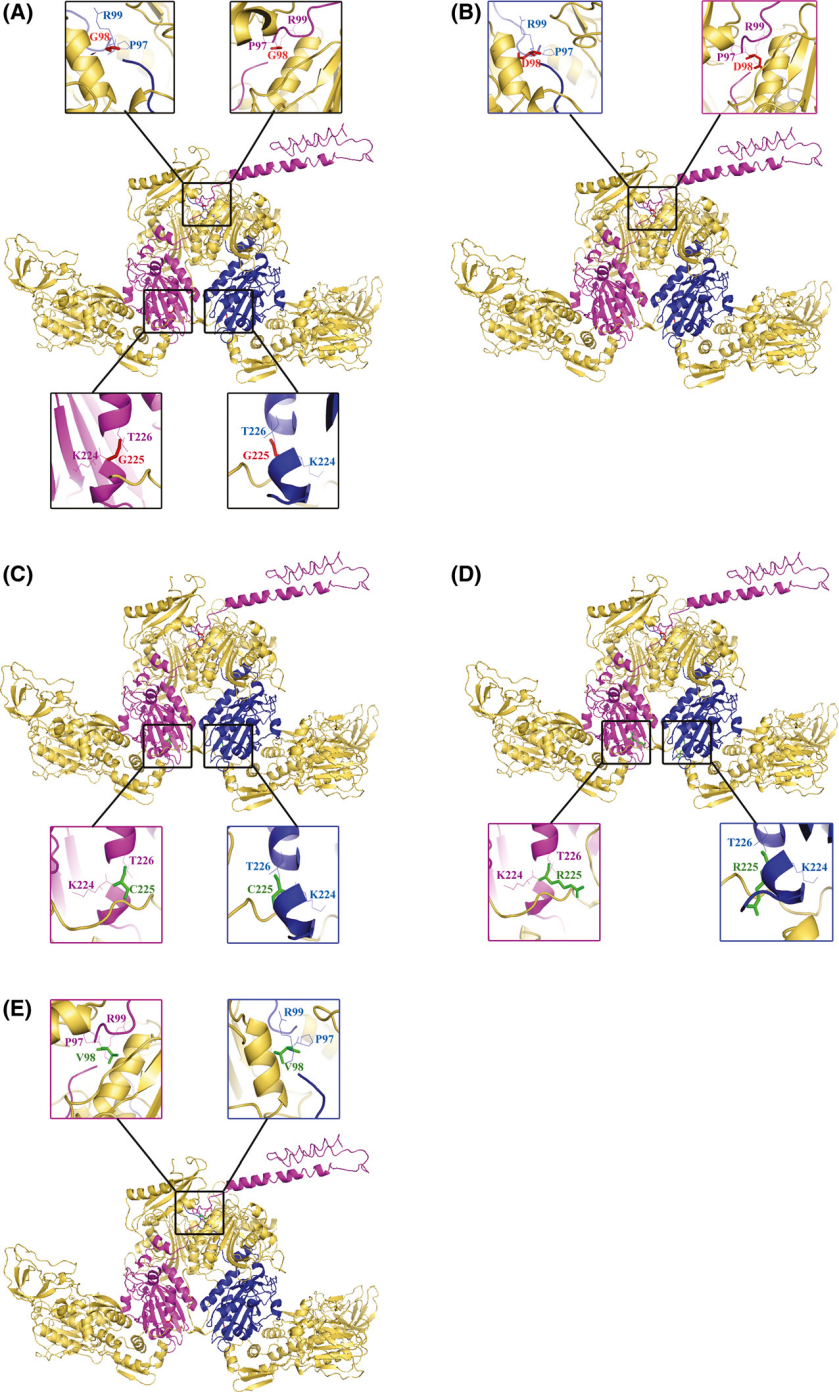
Figure showing relevant amino acids and mutations in the Crystal structure of PheRS enzyme: (A) WT PheRS; (B) PheRS from NP37 showing Gly_98_ to Asp_98_ mutation; (C) PheRS from TPM519 showing Gly98 to Asp98 and Gly_225_ to Cys_225_ mutations; (D) PheRS from TPM528 showing Gly_98_ to Asp_98_ and Gly_225_ to Arg_225_ mutations; (E) PheRS from TPM540 showing Asp_98_ to Val_98_ mutation. Note: A chain-blue, c chain-majenta, B and D-yellow orange color (template of *Escherichia coli* PheRS was adapted from Mermershtain et al., 2010) and pymolwin software was used for structure prediction of PheRS in all the cases.

**Figure 5:**
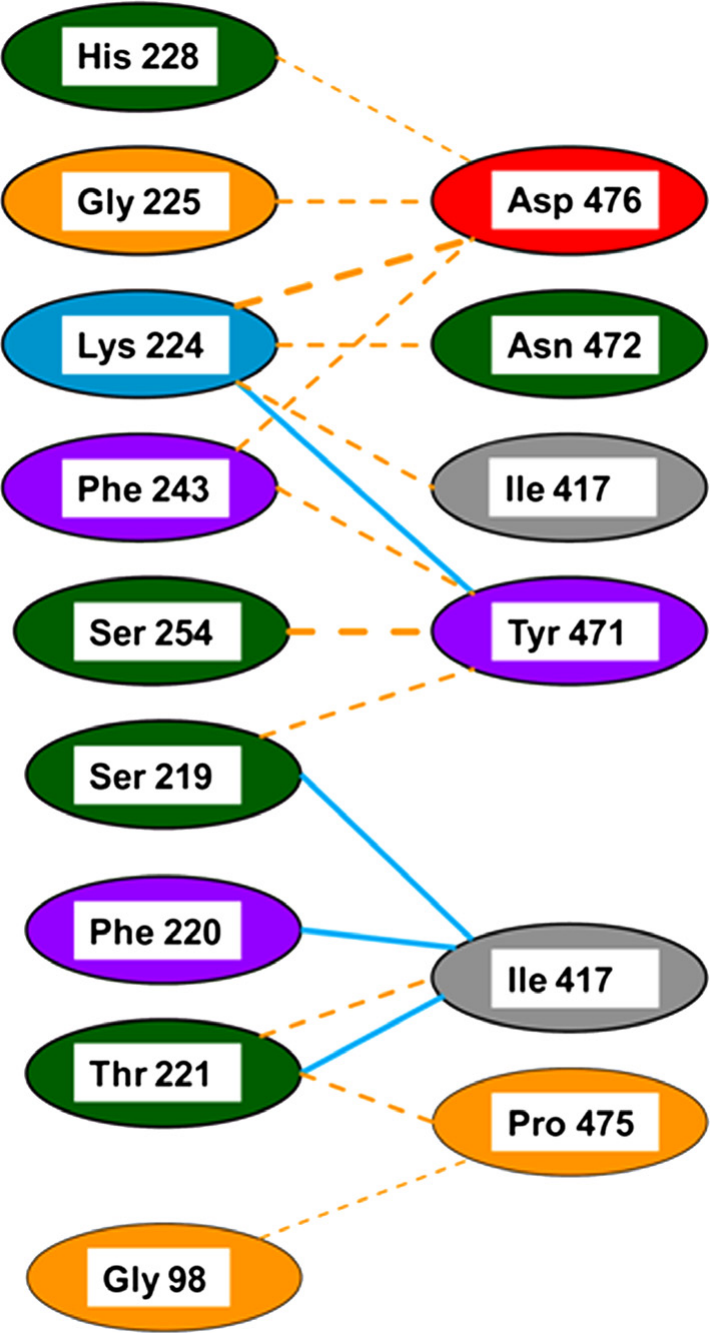
Schematic map of interaction between amino acids of C and D chains of PheRS enzyme. Note: This schema map was drawn based on the information available at http://www.ebi.ac.uk/thorntonsrv/databases/cgi-bin/pdbsum/GetPage.pl. It shows the indirect interaction between Gly_98_ and Gly_225_. The blue line indicates the hydrogen bond between the atoms and the orange striped line indicates the non-bonded contacts; the width of the striped line is proportional to the number of atomic contacts.

Mutated amino acid residues have been mapped on a three-dimensional structural model of EcPheRS (Fig. 4) This is based on the crystal structure of EcPheRS, determined by Mermershtain et al. (2011) (http://www.ebi.ac.uk/thorntonsrv/databases/cgi-bin/pdbsum/GetPage.pl). EcPheRS is a tetramer consisting of two *α* (A and C chains) and two *β* (B and D chains) subunits. Gly_225_ which is proposed as a mutational hot spot for intragenic suppressors of *pheS5* is found to be present in H5 helic (Phe_220_-Phe_235_ [FTNLK**G**TLHDFLRNFF]) of *α*1subunit and also in H9 helic (Phe_220_-Phe_234_ [FTNLK**G**TLHDF LRNF]) of *α*2subunit. We have given a figurative illustration (Fig. 5) implying the cross talk/possible interaction between the amino acids between *α*2 and *β*2 subunits of EcPheRS defined by the codon affected in *pheS5* lesion and the relevant amino acid change due to intragenic suppressors. But the same kind of residue interaction was not found between *α*1 and *β*1 subunits of EcPheRS (Mermershtain et al. 2011).

## Discussion

**Figure 6:**
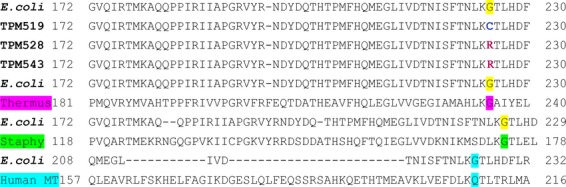
Multiple alignment between Chain A of PheRS Enzyme in relevant cases.

In interacting systems involved in macromolecular metabolism usually mutation(s) in one component can be compensated for by mutation(s) in other components. The mutation(s) capable of suppressing the phenotype of the original mutation are termed as suppressors. In fact, even today functional relation between two (or more) genes that is not possible to be deciphered by other means could very well emerge from suppressor analyses. While extragenic suppressors have been of immense value in identifying components of interacting systems involved in Macromolecular Metabolism, it is the intragenic suppressors that very much pave way for identifying amino acids that are crucial for the functional restoration of the mutant protein in appropriate growth conditions (Prelich 1999; Sujatha and Chatterji 2000; Hodgkin 2005; Mcclory et al. 2010). In this investigation, starting from one of the well-characterized temperature-sensitive translation-defective *pheS5* mutant NP37 defined by a G_293_ → A_293_ transition that is PheSG98D, we have reported the isolation and characterization of four pseudorevertants among 58 Ts^+^ derivatives. Of the four, in three Ts^+^ derivatives, the base position affected by the intragenic suppressor is virtually the same (base pair coordinate 673). To our surprise in two of the three cases the very base change itself is the same, G_673_ → C_673_ transversion (*pheS28* and *pheS43*). In one other case, the base change is defined by a G_673_ → T_673_ transversion (*pheS19*). Considering the fact that these Ts^+^ derivatives were isolated in six independent experiments and frequency of occurrence of suppressors’ of *pheS5* at the same coding position **G**GC(base pair coordinate 673) compelled us to make a model that this base pair coordinate 673 of *pheS* might define a hot spot for intragenic suppressors of *pheS5*. Therefore, the amino acid defined by the affected codon GGC → Gly_225_ should be of importance in keeping up the structural/functional integrity of PheS protein. Given in Figure 6 is the alignment of amino acids of the relevant position in PheRS from different species.

Intra and Extragenic suppressors have been reported by several groups in various genes affecting various functions. As early as in 1963, pioneering studies in *trp* operon by Helinski and Yanofsky revealed the suppression of *trpA46* mutation by intragenic suppressors in *trp* operon of *E. coli*. Miller et al. (1991) have reported about a mutant aminoacyl–tRNA synthetase that compensates for the mutation in the Major identity determinant of its tRNA. The intragenic suppressors of *ftsA13* Ts mutation have been reported by Robinson et al. (1991). Tavormina et al. (1996) studied the intragenic suppressor mutations for a class of elongation-defective and termination-proficient inviable *rpoB* alleles (that affect highly conserved residues) to know the regions of *β* that interact with each other. Davis et al. (1999) in an attempt to see the functional interaction between domains of Hsp70s, analyzed the mutations in the region encoding for the ATPase domain that were found to be intragenic suppressors of a lethal mutation (I485N) mapping to the peptide-binding domain of the mitochondrial Hsp70 Ssc1. Also the suppression of Ts phenotype in *dnaA508* mutant was observed by intragenic suppression (Eberle et al. 1989). In an interesting study Milija et al. (1999) have reported that mutations in tRNA synthetase genes (*alaS, argS, ileS* and *leuS*) that became resistant to even Gyrase inhibitor Novobiocin. Similarly, Kuo and Nakamoto (2000) showed the intragenic and extragenic suppression of the *E. coli* ATP synthase subunit *a* mutation defined by Gly-213 to Asn change and their study led to an understanding of functional interactions between residues in the proton transport site. Klein and Georgopoulos (2001), have reported the isolation and characterization of Temperature-insensitive derivatives of *groEL44* Ts mutant and found that 40/46 revertants were due to intragenic suppressors. The suppressors were shown to be defective in different amino acid substitution. In fact, it is these suppressor analyses that throw more light on the enhancement of cochaperon binding with Gp31 of T4. In a different report Shiomi et al. (2002) have reported the isolation of six independent intragenic suppressors of chemoreceptor Tar (W550A) mutant of *E. coli*. These suppressors were subdivided into two classes. Tar carrying class I suppressors and class II suppressors. It is this study that helped them to reinforce the importance of xWxxF motif and led them to suggest that the motif does not have to be located at the extreme of carboxy terminus of receptor. Rokop and Grossman (2009) have reported the isolation of intra and extragenic suppressors of Temperature-sensitive mutants in genes *dnaD* and *dnaB* involved in DNA Replication initiation in *Bacillus subtilis*. Their study has given new insights into structure–function relationship in DnaD and DnaB and interaction between both.

From this laboratory also intra and extragenic suppressors for different class of mutants affecting different functions in *E. coli* have been isolated (Vidya et al. 2006; and references cited therein). To be more specific, *fitA24* and *fitB* alleles were isolated as suppressors for the originally isolated Temperature-sensitive Transcription-defective *fitA76* mutant and suppression of transcription defect was found to be in an allele-specific manner (*fitA76- fitA24*; *fitA76*- *fitB*; *fitA24*- *fitB*), which strongly supported the involvement of Fit factors in transcription (Dass and Jayaraman 1985a,b; Munavar and Jayaraman 1987, 1993; Munavar et al. 1993; Ramalingam et al. 1999). Results of Munavar et al. (2005) have paved way for the understanding of the actual mechanism behind elicitation of Alp^+^ phenotype, which emerged from the analyses of mutations that suppress *lon* phenotype of *E. coli*. Singaravelan et al. (2010) have shown that the classical amber suppressor allele of *E. coli supE44* is an allele of *glnX* and this amber suppressor can also suppress even *ochre* and *opal* nonsense mutations to appreciable degree but only in stationary phase. Recently Shanmughapriya and Munavar (2012) have reported the suppression, specifically of MMC^S^ phenotype of *lexA3* mutant by a combination of specific *rpoB87-gyrA87* mutations. It has been demonstrated that this unconventional DNA repair originally called ‘SIR’ (referring to SOS Independent DNA Repair) elicited by *rpoB87-gyrA87* mutations stems from expression of *uvrB* in *lexA3* mutant. These *rif nal* alleles allow expression of *uvrB* but not *sulA* in otherwise SOS noninducible *lexA3* strain, which is believed to be unorthodox.

As could be seen from the results presented herein, the complementation of Temperature-sensitive phenotype due to *pheS5* by Plasmid clones pTPMS519 and pTPMS519A (bearing *pheS5-pheS19* alleles) and Plasmid clones pTPMS528 and pTPMS528A (bearing *pheS5-pheS28* alleles) in NP37 genetic background is poor. This could be perhaps due to the fact that the active *α* subunit of EcPheRS (*E. coli* PheRS) encoded by the *pheS5-pheS19* alleles and *pheS5-pheS28* alleles, which were functional in *cis* could not form such active complex in *trans* due to the hindrance by the mutant PheS protein, encoded by the *pheS5* allele in the chromosome. Our results make us to infer that excess of double mutant protein may not be good for survival of cells at 42°C. Our suppressor analysis compels us to make a model that there could be cross talk/interaction between Gly_98_ and Gly_225_ amino acids. Moreover, on the basis of extensive complementation analyses and the results reported herein, we propose that putative Alternate Promoter of *pheST* operon originally identified by Kamalakar (2006), from this laboratory could indeed help in better transcription of *pheS* gene when compared to that of Native promoter. This is because NP37 *pps::Tn10 recA::cam*/pTPMS^+^A shows better growth than NP37 *pps::Tn10 recA::cam*/pTPMS^+^. We further believe and postulate that *pheS* alleles present in the plasmid and those in the chromosome might play a critical role in determining the extent of restoration of PheRS enzyme activity, which truly reflects in the extent of complementation. Clones bearing just *pheS5* allele alone did not complement *pheS5* Ts phenotype regardless of presence of either Native promoter or Alternate Promoter. We do believe that to make firm conclusion about the veracity regarding the strength of the Alternate Promoter, we need to do further experiments and these are currently underway. Considering the vast majority of the literature pertaining to Informational suppression and the vital roles of such studies play in inferring the interaction between the components or domains or amino acid with in a protein, we believe that our proposal that G673 of *pheS* defining a hot spot for intragenic suppressors of *pheS5* could be of immense importance.

## Conclusion

In this investigation, by way of systematically analyzing a collection of temperature-insensitive derivatives of *pheS5*Ts mutant, we have clearly shown, perhaps for the first time, that the intragenic suppressors of *pheS5* define a hot spot (Base-pair coordinate 673 of *pheS*). This study clearly implies the importance of amino acid Gly_225_ in structural/functional integrity of PheS and hence PheRS enzyme. Considering the multifaceted nature of Aminoacyl-tRNA synthetases (Hausmann and Ibba 2008; Smirnova et al. 2012) and their crucial role in cellular functions (Kast et al. 1992; Safro et al. 2000; Ling et al. 2009). We do believe that our work presented herein will throw more light on EcPheRS and would help to gain further insights into the role of different domains. We also propose that this investigation will provide new directions to understand the effect of different antibacterial agents which inhibit Bacterial Translational fidelity.

## Supporting Information

**Table S1.** List of Bacterial strains (derivatives of *E.coli* K12) used in this study. **Table S2.** Plasmid vectors/clones, used/constructed in this study.
