# Supplementary material for: G673 could be a novel mutational hot spot for intragenic suppressors of pheS5 lesion in Escherichia coli
Source: Microbiologyopen. 2014 May 8;3(3):369–82. doi: 10.1002/mbo3.161 (PMC4082710; doi:10.1002/mbo3.161)
Supplement: Supplementary file 1 — Table S1. List of Bacterial strains (derivatives of E.coli K12) used in this study. Table S2. Plasmid vectors/clones, used/constructed in this study. [file mbo30003-0369-sd1.docx]

**Table S1 List of Bacterial strains (derivatives of *E.coli* K12) used in this study**

| **Strain** | **Genotype**  **(Relevant markers only)** | **Source/Construction**  **/Reference** |
| --- | --- | --- |
| MG1655  NP37 | F^-^ *λ^-^ rph-1*  HfrC *pheS5* | CGSC, USA  Lab collection |
| NP37 *pps::Tn10* | *pps::Tn10* derivative of NP37 | This work |
| Ts^+^ derivative 1 | Temperature insensitive derivative 1 isolated from NP37 *pps::Tn10* (True revertant, *pheS^+^*) | This work |
| Ts^+^ derivative 2 | Temperature insensitive derivative 2 isolated from NP37 *pps::Tn10* (True revertant, *pheS^+^*) | This work |
| Ts^+^ derivative 3 | Temperature insensitive derivative 3 isolated from NP37 *pps::Tn10* (True revertant, *pheS^+^*) | This work |
| Ts^+^ derivative 37 | Temperature insensitive derivative 37 isolated from NP37 *pps::Tn10* (True revertant, *pheS^+^*) | This work |
| Ts^+^ derivative 50 | Temperature insensitive derivative 50 isolated from NP37 *pps::Tn10* (True revertant, *pheS^+^*) | This work |
| Ts^+^ derivative 55 | Temperature insensitive derivative 55 isolated from NP37 *pps::Tn10* (True revertant, *pheS^+^*) | This work |
| TPM519 | 19^th^ Ts^+^ derivative isolated from NP37 *pps::Tn10*; shown to bear  *pheS5*-*pheS19* alleles | This work |
| TPM528 | 28^th^ Ts^+^ derivative isolated from NP37 *pps::Tn10*;shown to bear  *pheS5*-*pheS28* alleles | This work |
| TPM543 | 43^rd^ Ts^+^ derivative isolated from NP37 *pps::Tn10*; shown to bear  *pheS5*-*pheS43* alleles | This work |
| TPM540 | 40^th^ Ts^+^ derivative isolated from NP37 *pps::Tn10*; shown to bear  *pheS40* allele | This work |
| BW11334/pBW120 | F',*Δ(argF-lac)169recA1907::cat-aadAthi-1Δ(phnP-phnD)3330(phnC?)creC510* F128-Tn10-11 pBW120 | CGSC, USA |
| MC4100 | F^-^ *Δ(argF-lac)169 relA1 rpsL150* | Lab collection |
| NP37 *pps::Tn10 recA::cam* | *recA::cam* derivative of NP37*pps::Tn10* | This work |
| MC4100 *recA::cam* | *recA::cam* derivative of MC4100 | This work |
| NP37 *pps::Tn10 recA::cam/* pTPMS519 | NP37 *pps::Tn10 recA::cam* bearing pTPMS519 plasmid harbouring *pheS5-pheS19* alleles with NP and AP | This work |
| NP37 *pps::Tn10 recA::cam/* pTPMS528 | NP37 *pps::Tn10 recA::cam* bearing pTPMS528 plasmid harbouring *pheS5-pheS28* alleles with NP and AP | This work |
| NP37 *pps::Tn10 recA::cam/* pTPMS5 | NP37 *pps::Tn10 recA::cam* bearing pTPMS5 plasmid harbouring *pheS5* allele with NP and AP | This work |
| NP37 *pps::Tn10 recA::cam/* pTPMS^+^ | NP37 *pps::Tn10 recA::cam* bearing pTPMS^+^ plasmid harbouring *pheS^+^* allele with NP and AP | This work |
| NP37 *pps::Tn10 recA::cam/* pTPMS519A | NP37 *pps::Tn10 recA::cam* bearing pTPMS519A plasmid harbouring *pheS5-pheS19* alleles with AP | This work |
| NP37 *pps::Tn10 recA::cam/* pTPMS528A | NP37 *pps::Tn10 recA::cam* bearing pTPMS528A plasmid harbouring *pheS5-pheS28* alleles with AP | This work |
| NP37 *pps::Tn10 recA::cam/* pTPMS5A | NP37 *pps::Tn10 recA::cam* bearing pTPMS5A plasmid harbouring *pheS5* allele with AP | This work |
| NP37 *pps::Tn10 recA::cam/* pTPMS^+^A | NP37 *pps::Tn10 recA::cam* bearing pTPMS^+^ plasmid harbouring *pheS^+^* allele with AP | This work |

Note: NP denotes Native promoter of *pheST* operon; AP denotes Alternate promoter of *pheST* operon; For other details see text

**Table S2 Plasmid vectors/clones, used/constructed in this study**

| **Name of the**  **Plasmid**  **/Clone** | **Relevant genes/allele(s)/ markers present** | **Source/**  **Construction**  **/Reference** |
| --- | --- | --- |
| pBR322 | Amp^R^ Tet^R^ | Lab collection |
| pTPMS519 | Derivative of pBR322 harbouring ~1.5kb fragment bearing *pheS5-pheS19* alleles with NP and AP, Amp^R^ Tet^S^ | This work |
| pTPMS528 | Derivative of pBR322 harbouring ~1.5kb fragment bearing *pheS5-pheS28* alleles with NP and AP, Amp^R^Tet^S^ | This work |
| pTPMS5 | Derivative of pBR322 harbouring ~1.5kb fragment bearing *pheS5* allele with NP and AP, Amp^R^Tet^S^ | This work |
| pTPMS^+^ | Derivative of pBR322 harbouring ~1.5kb fragment bearing *pheS^+^* allele with NP and AP, Amp^R^Tet^S^ | This work |
| pTPMS519A | Derivative of pBR322 harbouring ~1.5kb fragment bearing *pheS5-pheS19* alleles with AP, Amp^R^Tet^S^ | This work |
| pTPMS528A | Derivative of pBR322 harbouring ~1.5kb fragment bearing *pheS5-pheS28* alleles with AP, Amp^R^Tet^S^ | This work |
| pTPMS5A | Derivative of pBR322 harbouring ~1.5kb fragment bearing *pheS5* allele with AP, Amp^R^Tet^S^ | This work |
| pTPMS^+^A | Derivative of pBR322 harbouring ~1.5kb fragment bearing *pheS^+^* allele with AP, Amp^R^Tet^S^ | This work |
